# Silicon Deposition and Phytolith Morphological Variation in Culm Sheaths of *Dendrocalamus brandisii* at Different Growth Stages

**DOI:** 10.3390/plants14060841

**Published:** 2025-03-07

**Authors:** Siyuan Duan, Maobiao Li, Dongbo Xie, Rui Xu, Shuguang Wang, Changming Wang, Hui Zhan

**Affiliations:** 1 College of Forestry, Southwest Forestry University, Kunming 650224, China; duansiyuan2@swfu.edu.cn (S.D.); benjiamin@swfu.edu.cn (M.L.); apfc@swfu.edu.cn (D.X.); xurui@swfu.edu.cn (R.X.); stevenwang1979@126.com (S.W.); 2 Key Laboratory for Sympodial Bamboo Research, Southwest Forestry University, Kunming 650224, China

**Keywords:** culm sheath, phytolith, morphology, variation, growth stage, *Dendrocalamus brandisii*

## Abstract

Bamboo is an efficient silicon accumulator with diverse phytolith morphotypes and composition. The bamboo culm sheath, traditionally considered as a modified leaf, plays a key role in bamboo taxonomy and provides significant mechanical and physiological support for shoot development, but its silicon deposition and phytolith morphological variation remain underexplored. We investigated silicon variation and phytolith morphology in *D. brandisii* culm sheaths at different growth stages. The results showed that silicon deposition in *D. brandisii* culm sheaths at different growth stages was comparable to foliage leaves but significantly greater than branches as in previous research. Phytolith concentration in the culm sheath blades of *D. brandisii* was higher, associated with their greater silicon content than the sheath bodies. Silicon precipitated and phytoliths were produced as the culm sheath matured. Silicon and phytolith contents were significantly greater in upper culm sheath bodies. *D. brandisii* culm sheaths were characterized by a high proportion of rondel phytoliths, whereas bilobate and bulliform flabellate phytoliths were not observed. Circular and saddle phytoliths accounted for a very low proportion. Stomata phytoliths were abundant in culm sheaths at the shooting stage and increased with sheath maturation, which supported the transpiration, respiration and photosynthesis in culm sheaths of the shoots. Elongate and acute phytoliths were extremely abundant in *D. brandisii* culm sheaths and increased with sheath maturation, which enhanced the mechanical and protective role of the culm. The phytolith morphotypes in *D. brandisii* culm sheaths did not demonstrate a specific trend with sheath position. Saddle phytoliths showed insignificant variation in *D. brandisii* culm sheaths.

## 1. Introduction

Sheath is an important organ in Poaceae plants with special morphological characteristics. There are primarily three types of bamboo sheaths, viz. culm sheath, rhizome sheath and foliage leaf sheath [1]. The culm sheath plays a key role in bamboo taxonomy, due to the long reproductive period and difficulties in obtaining its reproductive organs [2]. Bamboo shoots are usually wrapped tightly in partly overlapping sheaths to protect and support their elongating nodes and internodes, providing significant mechanical support when young culms develop [3]. The culm sheath is also a major transpiration, respiration and photosynthesis organ of bamboo shoots [4,5]. It plays an important role in the rapid growth of bamboo shoots by regulating water transport and pressure as well as sugar transport and metabolism in the internodes [4,5]. Bamboo culm sheaths are green or in some cases other colors before turning brown, and wrap the mature culms for some time [6]. When the internode elongation completes, the sheaths dry out and eventually fall off.

**Figure 1:**
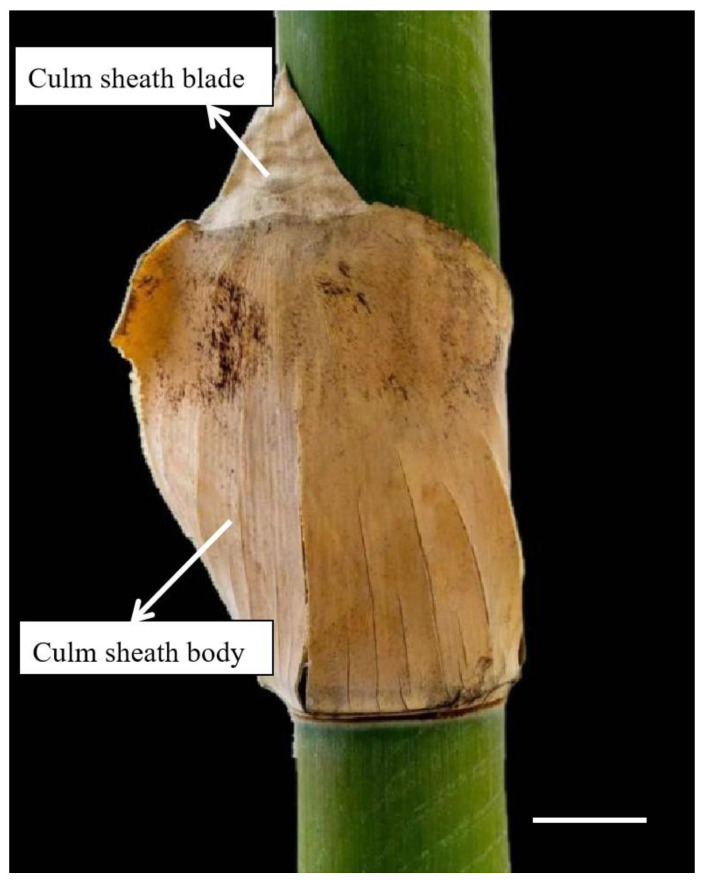
Culm sheath of *Dendrocalamus brandisii* (bar = 10 cm).

The blade usually grows at the culm sheath’s tip (Figure 1). The bamboo culm sheath is considered a modified leaf because of its unique leaf-like shape [6,7]. However, Wang [1] determined that bamboo foliage leaf has a petiole joining the blade to the sheath body, but such a petiole is absent in the culm sheath. The petiole is considered as an important distinction between bamboo and other grasses. In addition, there is a midrib in the foliage leaf blade, which does not exist in the culm sheath. Moreover, through the anatomical structure observation on *Fargesia yunnanensis* culm sheath, it was reported that culm sheath is more likely a modified branch, rather than a modified leaf [1].

Phytoliths are microscopic amorphous silica structures produced in and between plant cells by precipitation and polymerization of silica absorbed by plant roots/rootlets from soils [8]. Phytoliths are produced in a variety of plants, and their morphology varies greatly among plants as well as in different parts of the same plant. Phytoliths can be used to identify a particular genus or species based on shape, size, and other anatomical features [9,10]. As an efficient silicon accumulator, bamboo has a high phytolith concentration. Phytoliths in several bamboo species from the genera *Bambusa*, *Dendrocalamopsis*, *Dendrocalamus* and *Ferrocalamus* were previously studied, showing that saddle and bilobate phytoliths accounted for a high percentage in bamboo foliage leaves, whereas bulliform and elongate phytoliths had a moderate proportion or were rare in the grass family [11,12,13,14,15,16]. *Bambusa* had a relatively higher percentage of oblong concave saddles, bulliform cells and rondels than *Dendrocalamopsis* and *Dendrocalamus*, while *Dendrocalamus* had a relatively higher percentage of bilobates than the other two [11]. Oblong concave saddles exhibited taxonomical value both at the subfamily and genus levels [11]. To date, most of the previous studies focused primarily on the phytoliths in bamboo foliage leaves, given that they had significantly higher silicon content and a greater diversity of phytolith morphotypes than any other organs [11,12,13,14,15]. However, phytolith concentration and morphology in bamboo foliage leaves were influenced by factors such as nursery methods and environmental conditions [12,13,14,15], which to some extent, affected the accuracy of leaf phytoliths in bamboo classification. In fact, specific organs of bamboo produced specific morphotypes of phytoliths [12,16]. Since bamboo culm sheath was considered a modified leaf, we hypothesized that the phytolith morphotypes and composition in bamboo culm sheath might be similar to that of the foliage leaf, and it might be a supplementary indicator for bamboo identification. However, the phytolith morphology of bamboo culm sheaths has been barely researched.

*Dendrocalamus brandisii*, a sympodial bamboo species, occurs in the wild primarily in the tropical and subtropical regions of China and southeast Asia, including Myanmar, Laos, Vietnam, and Thailand. The largest plantations of *D. brandisii* were established in southern and southwestern Yunnan of China [13]. The *D. brandisii* sheath is reddish brown or bright yellow and among the largest of the bamboo species. It is leathery and glabrous with longitudinal strips on the epidermis [17]. The auricle is small with oral setae of around 1 cm [17]. Zhan et al. [12] analyzed the phytolith morphology in different organs of *D. brandisii*, but their research on bamboo culm sheaths was insufficient, e.g., they did not determine the phytolith morphotypes in different positions of culm sheaths at different growth stages.

This study determined the silicon variation and phytolith morphology in *D. brandisii* culm sheaths at different growth stages and explored phytolith changes with culm sheath growth and function, providing evidence for the relation between bamboo culm sheath (modified leaf) and foliage leaf in terms of phytolith morphology. It also provided a theoretical basis and references for elucidating the mechanical and physiological functions of bamboo sheath.

## 2. Results

### 2.1. Silicon and Phytolith Content in D. brandisii Culm Sheath Blades at Different Growth Stages

Silicon distribution in the culm sheath blades of *D. brandisii* at different growth stages was unequal and increased in the order of shooting stage (25.53 g · kg^−1^) < culm stage (127.18 g · kg^−1^) < shedding stage (150.55 g·kg^−1^). During culm sheath maturation, the silicon in sheath blades rapidly accumulated (Table 1). 

Similarly to the trend observed for silicon, the phytolith content in *D. brandisii* sheath blades also increased with culm sheath maturation, and the sheath blades at the shedding stage (103.44 ± 3.03 g·kg^−1^) showed significantly higher phytolith content than that at culm (40.38 g · kg^−1^) and shooting stages (32.92 g · kg^−1^) (Table 1).

### 2.2. Silicon and Phytolith Content in the D. brandisii Culm Sheath Bodies at Different Growth Stages

It was noted that the silicon and phytolith average in *D. brandisii* culm sheath bodies was less than that in culm sheath blades (Table 1 and Table 2).

The silicon content in culm sheath bodies increased with the sheath maturation and reached the maximum value at the culm stage (34.41 g·kg^−1^) (Table 2). The sheath bodies at the culm stage had significantly higher silicon content than those at shedding and shooting stages. The silicon content in the *D. brandisii* culm sheath bodies also varied with different positions (Table 2). It was observed that at the shedding stage, the upper part of the culm sheath bodies had the greatest silicon content. However, at the shooting and culm stages, silicon in different positions of sheath bodies varied insignificantly.

The phytolith content in the culm sheath bodies of *D. brandisii* increased with the sheath maturation and reached the maximum value at the shedding stage (32.25 g·kg^−1^), which was significantly higher than that at the culm and shooting stages (Table 2). It was noted that the upper part had a significantly lower phytolith content at the shooting stage, whereas it had a higher phytolith content at the shedding stage.

### 2.3. Phytolith Morphotypes and Their Variation in the D. brandisii Culm Sheath Blades at Different Growth Stages

**Figure 2:**
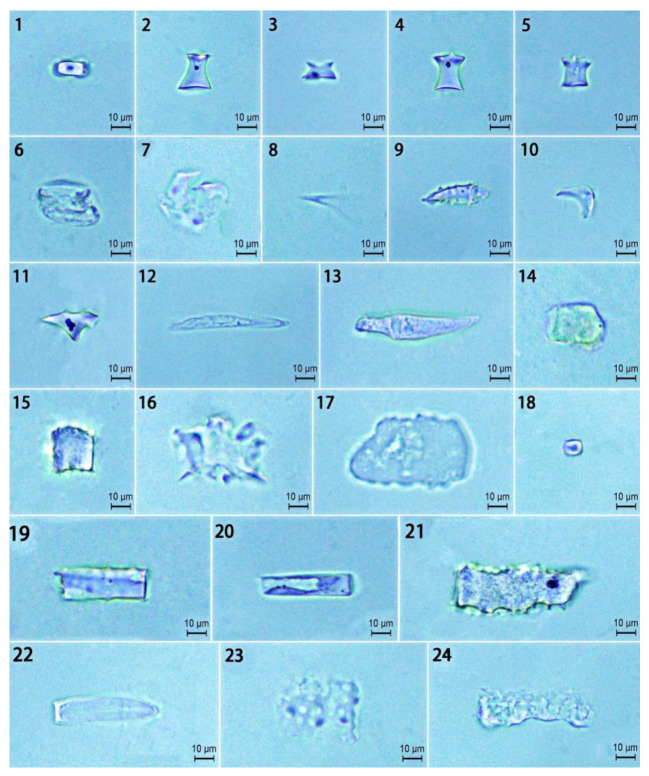
The phytolith morphotypes in the *D. brandisii* culm sheath blades (bars = 10 μm). 1. Saddle. 2. Ruffle top rondel. 3. Two-spiked rondels. 4. Three-spiked rondels. 5. Four-spiked rondels. 6 and 7. Stomata. 8. Acute. 9. Echinate acute. 10 and 11. Acute. 12. Extended acute. 13. Granulate extended acute. 14. Blocky. 15. Echinate blocky. 16. Plicate blocky. 17. Blocky (rectangular). 18. Circular. 19. Entire elongate. 20. Scrobiculate elongate. 21. Echinate elongate. 22. Bulbous elongate. 23. Scrobiculate elongate. 24. Tuberculate elongate.

**Figure 3:**
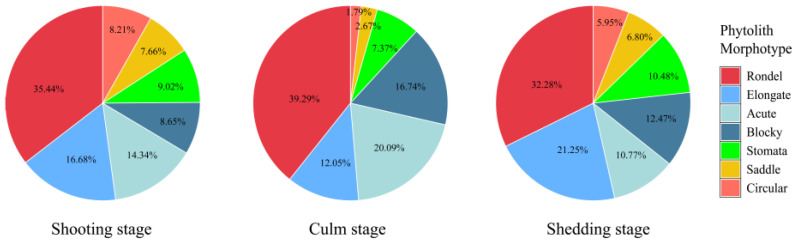
The proportion of phytolith morphotypes in the *D. brandisii* culm sheath blades.

The phytolith morphotypes in the *D. brandisii* culm sheath blades were observed and counted using the classification systems of International Code for Phytolith Nomenclature (ICPN 1.0) [18] and ICPN 2.0 [19]. Phytolith morphotypes in the *D. brandisii* culm sheath blades at different growth stages were identical and classified into seven morphotypes (24 sub-morphotypes), including rondel (ruffle top rondel, two-spiked rondel, three-spiked rondels, four-spiked rondels), elongate (entire elongate, scrobiculate elongate, echinate elongate, bulbous elongate, scrobiculate elongate, tuberculate elongate), acute (echinate acute, reniform conical, extended acute, granulate extended acute), blocky (square, rectangular), stomata, saddle and circular (Figure 2). The rondel phytoliths were the dominant phytolith morphotype in *D. brandisii* culm sheath blades at different growth stages, while saddle and circular phytoliths occurred at relatively low frequency (Figure 3).

We used all seven observed phytolith morphotypes to analyze their variations in the *D. brandisii* culm sheath blades and found that with maturing of *D. brandisii* sheaths, the dominant phytolith morphotypes showed some difference. The rondel phytoliths accounted for the highest proportion at different sheath stages, which varied in the order of culm stage (39.29%) > shooting stage (35.44%) > shedding stage (32.28%). Besides rondel, two other phytolith morphotypes (elongate, acute) also had a high proportion (>10%) at the shooting stage. In maturing sheaths, the proportion of blocky phytoliths exhibited an increasing trend attaining >10% at culm and shedding stages. The stomata phytoliths also demonstrated an increasing trend and attained 10.48% at the shedding stage. Therefore, at the shedding stage, except for the phytolith morphotypes of rondel, elongate, acute and blocky, stomata were included in the phytolith morphotypes with occurrence above 10%. However, saddle and circular phytoliths occurred at relatively low frequency (<10%) in the *D. brandisii* culm sheath blades at different growth stages, which further decreased at the culm stage (<3%) (Figure 3).

### 2.4. Phytolith Morphotypes and Their Variation in the D. brandisii Culm Sheath Bodies at Different Growth Stages

**Figure 4:**
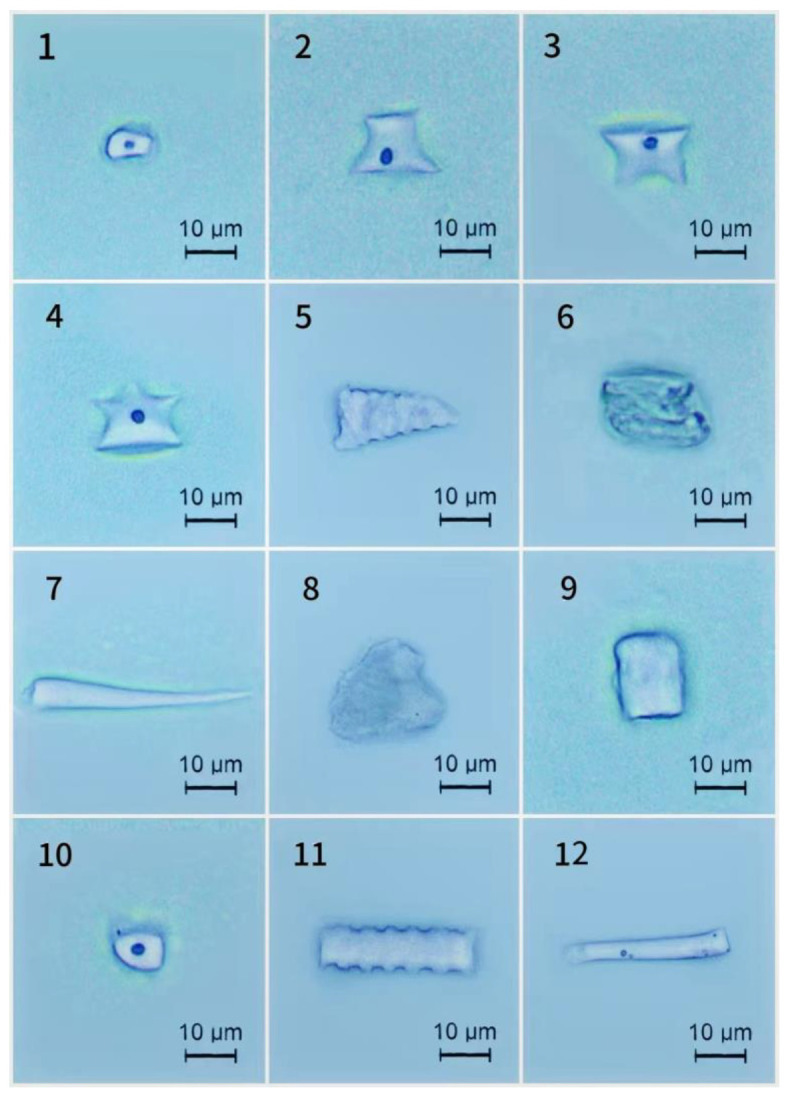
The phytolith morphotypes in the *D. brandisii* culm sheath bodies (Bars = 10 μm). 1. Saddle. 2. Ruffle top rondel. 3. Two-spiked rondel. 4. Three-spiked rondel. 5. Echinate acute. 6. Stomata. 7. Extended acute. 8. Blocky. 9. Echinate blocky. 10. Circular. 11. Echinate elongate. 12. Scrobiculate elongate.

The phytolith morphotypes in the culm sheath bodies were less diversified than the blades of *D. brandisii*. Seven broad phytolith morphotypes (twelve sub-morphotypes) were observed in the *D. brandisii* culm sheath bodies, viz. rondel (ruffle top rondel, two-spiked rondels, three-spiked rondels), elongate (echinate elongate, scrobiculate elongate), blocky (echinate square), acute (echinate acute, extended acute), saddle, stomata and circular (Figure 4).

**Figure 5:**
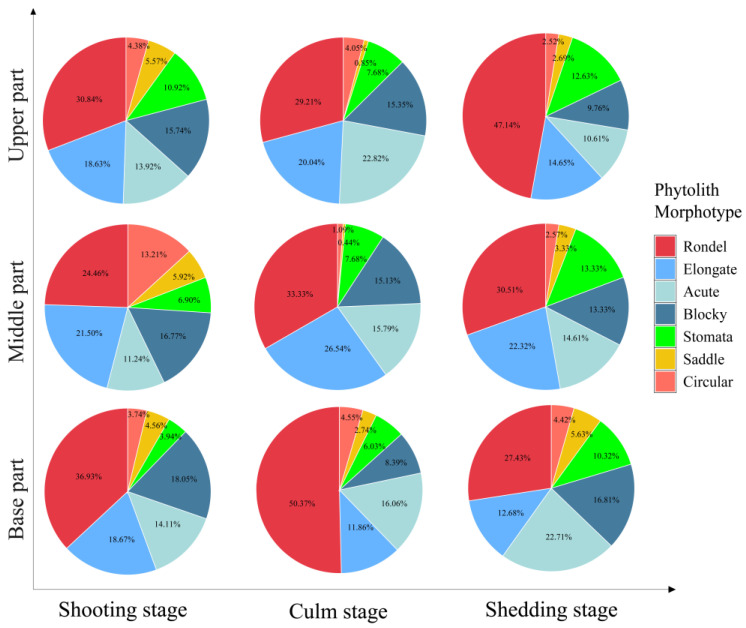
The proportion of phytolith morphotypes in the *D. brandisii* culm sheath bodies.

Similar to the culm sheath blades, the culm sheath bodies of *D. brandisii* were characterized by high proportions of rondel (>24%), elongate and acute phytoliths (>10%), whereas the saddle and circular phytoliths occurred at relatively low frequency. The proportion of different phytolith morphotypes in the culm sheath bodies varied with different growth stages and positions (Figure 5).

The rondel phytoliths ranked the highest proportion in the culm sheath bodies. Its proportion at shooting and culm stages decreased from the base to the upper of culm sheath bodies, whereas at the shedding stage, it demonstrated an increasing trend and the upper part had the highest rondel phytolith occurrence. During culm sheath maturation, rondel phytoliths in the base part increased from 36.93% at the shooting stage to 50.37% at the culm stage but decreased to 29.21% at the shedding stage. The rondel phytoliths in the middle part also showed a similar increasing trend. However, rondel phytoliths in the upper part slightly decreased from 30.84% at the shooting stage to 24.46% at the culm stage, but dramatically increased to 47% at the shedding stage. The proportion of rondel phytoliths was the highest in the base sheath bodies at the culm stage (50.37%) (Figure 5).

The elongate phytoliths represented the second highest proportion in the culm sheath bodies. At different growth stages, similar trends were observed that increased from the base to the middle part but then decreased in the upper parts. Therefore, it could be noted that the middle sheath bodies had the highest elongate phytolith occurrence. During sheath maturation, the elongate phytoliths in the base part demonstrated a decreasing trend, but in the middle and upper parts, it showed an increasing trend and reached the highest occurrence at culm stage. The proportion of elongate phytoliths was the highest in the middle sheath bodies at the culm stage (26.54%) (Figure 5).

The highest blocky phytolith occurrence was observed in the base part at the shooting stage (18.05%). However, its proportion exhibited a decreasing trend from the base to the upper part, which was similar to that at shedding stage. During sheath maturation, its proportion in different parts exhibited a decreasing trend (Figure 5).

The acute phytoliths accounted for 14.11% in the base part of culm sheath bodies at the shooting stage, which decreased to 11.24% in the middle part, but reached to 13.92% in the top part. It could be seen that more acute phytoliths were observed in the upper sheath bodies at the culm stage, while at the shedding stage, it decreased from the base to the upper sheath bodies. During sheath maturation, the acute phytoliths in the base sheath bodies demonstrated an increasing trend. In the middle and upper parts, they firstly showed an increasing trend and reached the highest rate at the culm stage, but then demonstrated a decreasing trend at the shedding stage (Figure 5).

Stomata phytoliths increased with sheath maturation and the sheath bodies at the shedding stage had a higher rate of stomata phytoliths than that at the shooting and culm stages. Additionally, it was noted that the stomata phytoliths increased with the height of culm sheath. More stomata phytoliths occurred in the upper sheath bodies (Figure 5).

With sheath maturation, saddle phytoliths in the base sheath bodies exhibited an increasing trend, whereas a decreasing trend was observed in the middle and upper parts. The proportion of saddle phytoliths in the culm sheath bodies at the shooting stage increased with the height of culm sheaths, while it decreased at the culm and shedding stages (Figure 5).

The proportion of circular phytoliths did not show specific variation trends either with the sheath stages or positions. In the base sheath bodies, it slightly increased to 4.55% at the culm stage but decreased to 4.42% at the shedding stage. However, in the middle part, it dramatically decreased from the shooting stage (13.21%) to culm stage (1.09%), but slightly increased to 2.74% at the shedding stage. In the upper part, the proportion of the circular phytoliths at the shedding stage was lower than that at the shooting and culm stages (Figure 5).

### 2.5. Phytolith Size and Its Variation in the D. brandisii Culm Sheath Blades at Different Growth Stages

With the sheath maturation, the size of rondel and circular phytoliths in the sheath blades of *D. brandisii* increased first and then decreased slightly. The rondel and circular phytoliths were bigger at the culm stage. However, the size of elongate and blocky phytoliths demonstrated an opposite trend that decreased first at the culm stage and then considerably increased at the shedding stage. Therefore, it could be seen that the length and width of elongate and blocky phytoliths in sheath blades reached the maximum at the shedding stage. The length and width of acute phytoliths in sheath blades at the shooting stage had the greatest value and with sheath maturation, it showed a downward trend. The stomata phytoliths at the culm stage were smaller than those at shooting and shedding stages. The length of saddle phytoliths increased dramatically from the shooting stage to the culm stage, but decreased again at shedding stage, while the saddle width did not vary significantly (Table S1).

### 2.6. Phytolith Size and Its Variation in D. brandisii Culm Sheath Bodies at Different Growth Stages

The size of different phytolith morphotypes in *D. brandisii* culm sheath bodies also varied with sheath maturity (Table S2). Generally, the size of rondel phytoliths exhibited a decreasing trend with sheath maturation. At the shooting stage, both the length and width of rondel phytoliths decreased from the base to the upper part of the sheaths, whereas at the culm stage, it demonstrated an increasing trend and the upper sheath bodies had the greatest size value. At the shedding stage, the length of rondel phytoliths increased from the base to the upper sheath bodies, whereas their width showed insignificant difference (Table S2).

During sheath maturation, the elongate phytoliths became bigger and attained the greatest size at the culm stage. Moreover, it was observed that they had the greatest length and width in the middle part, but the smallest values in the base part (Table S2). Similar to the elongate phytoliths, the blocky phytoliths attained the greatest size at the culm stage. In the base part, the blocky phytoliths were longer than those in the upper part at different growth stages. Moreover, the width of the blocky phytoliths in the base part was greater than the upper part, while their width at shooting and shedding stages did not vary significantly (Table S2). Acute phytoliths became thinner but longer with sheath maturation. Furthermore, it was noted that the acute phytoliths in the upper part of the culm sheath bodies were longer than those in the base part at different growth stages. At the shooting stage, the base and middle parts had wider acute phytoliths, while at the culm stage, the upper part had wider acute phytoliths (Table S2).

Both the length and width of stomata phytoliths exhibited a decreasing trend with sheath maturation. The sizes of the stomata phytoliths at the shedding stage were smaller than that at the shooting stage. Additionally, the length and width of stomata phytoliths in the culm sheath bodies at the shooting and culm stages decreased from the base part to the upper part. At the shedding stage, it demonstrated an opposite trend, where the upper part had larger stomata phytoliths (Table S2).

The size of saddle phytoliths did not vary significantly with the sheath maturation. It was noted that the upper part of culm sheath bodies at shooting and culm stages had bigger saddle phytoliths, while at the shedding stage, their length and width did not vary significantly with the positions (Table S2).

With the culm sheath maturation, the length of circular phytoliths increased, whereas the width decreased first at the culm stage and then increased significantly at the shedding stage. At the shooting stage, the base part had bigger circular phytoliths than the upper one. However, at culm and shedding stages, larger circular phytoliths were observed in the middle part of the sheath bodies (Table S2).

## 3. Discussion

### 3.1. Variation in Silicon and Phytolith Content in Culm Sheaths

The leaf of a bamboo plant is differentiated into two distinct morphotypes with different functions: culm sheath and foliage leaf [1]. The culm sheath predominantly envelops an internode and performs primarily a protective function. It safeguards the tender part of the lower internode while it is actively dividing and elongating, thereby playing a crucial role for bamboo growth [1]. The foliage leaf develops on the finer branches for photosynthesis [1]. Each form bears a blade at its apical end. From the perspective of organ evolution, foliage leaf sheath and culm sheath belong to the evolution of homologous organs [1]. The silicon content in the *D. brandisii* culm sheath blades at different growth stages was much higher than that in foliage leaf blades as reported from our previous study, at 18.98 g · kg^−1^ for *D. brandisii* young leaf blades, 25.58 g · kg^−1^ for mature leaf blades, and 28.12 g · kg^−1^ for old leaf blades, and also greater than the branches (2.89 g · kg^−1^) and culms (2.02 g · kg^−1^) [12]. The silicon content was also higher in the *D. brandisii* sheath blades at culm and shedding stages than in foliage leaf blades of other bamboo species, e.g., *D. giganteus* and *Ferrocalamus strictus* [15,16]. Previous studies have verified the mechanical functions of bamboo culm sheaths to protect bamboo shoots from damage of external force [6,20]. Bamboo culm sheath also plays physiological functions and significantly influences water and assimilate transport or the rapid growth of bamboo shoots [4,5]. Silicon absorbed by plant rootlets is transported in xylem precipitating mainly in tissues with high photosynthetic activity and transpiration [21,22]. In the tissues with photosynthetic activity, silicon polymerization and precipitation due to supersaturation by transpiration-driven water loss occur [22]. Silicon content in grass differed in uptake/transportation tissues from that in the tissues with transpiration function [23]. The culm sheath blade, with a thick cuticle but without apparent differentiation of mesophyll cells, was undertaking respiration and transpiration function, while the culm sheath body was transporting water and nutrients [5]. This might be the reason that the *D. brandisii* culm sheath blades had a higher phytolith concentration corresponding to their higher silicon content than the culm sheath bodies. The silicon content in the culm sheaths of *D. brandisii* increased as they matured, and a higher phytolith concentration was found in the sheaths at culm and shedding stages, indicating that the silicon was precipitating and phytoliths were continuously produced with the maturing of sheaths. The silicon content in various organs of bamboo was positively correlated with bamboo age [16]. Generally, silicon content in the young tissues of bamboo was lower than that in mature tissues, as the growth and elongation of young tissues were not complete; therefore, the silicon deposition was insufficient, resulting in a significantly lower phytolith concentration in sheaths at the shooting stage. Moreover, the silicon and phytolith content in culm sheath bodies varied with its positions. In our study, it was observed that the upper sheath bodies at the shooting stage had obviously lower silicon and phytolith content, whereas at the culm and shedding stages, the upper sheath bodies had significantly greater silicon and phytolith content. This indicated that the silicon in *D. brandisii* culm sheaths exhibited an upward flow of transportation and accumulation, and the upper sheath bodies matured first.

### 3.2. Variation In Phytolith Morphotypes and Composition in Culm Sheaths

In plants, phytoliths are silica microbodies produced from silica deposits in and around cells [24]. The phytoliths “replicate” the original morphology of the plant cell and record the characteristics of the plant cell from which it originated [24]. Phytolith morphology in foliage leaves exhibits taxonomical value both at the subfamily and genus levels [11,24,25,26]. *D. brandisii* foliage leaf blades had a great diversity of phytolith morphotypes, which were grouped into eight morphotypes, including round, elliptical, rectangular, saddle, bilobate, elongate, fan and acute [12]. Saddle and bilobate phytoliths accounted for over 80% of all phytoliths in *D. brandisii* foliage leaves [12]. This was consistent with the findings from Gu et al. on phytoliths of 26 bamboo species that bamboo foliage leaves were characterized by a high frequency of bilobate and saddle phytoliths [11]. However, bilobate, saddle, fan and acute phytoliths were reported in small numbers or barely detected in matured branches of *D. brandisii*, *D. giganteus* and *Ferrocalamus strictus* [12,15,16]. In the present study, seven phytolith morphotypes were observed in the *D. brandisii* culm sheaths, including rondel, stomata, acute, blocky, circular, elongate and saddle. Bilobate phytoliths were not detected and the saddle phytoliths occurred at relatively low frequency. This was the significant difference in the phytolith morphotypes between the bamboo foliage leaf and culm sheath. Additionally, the variation in bilobate morphology can be related to environmental factors, especially moisture [27]. Usually, bilobates with scooped ends arranged in parallel over or between veins are typical of the Oryzoideae (Ehartoideae) sub-family [9]. In contrast to the Panicoideae sub-family, *Oryza* bilobates exhibit wider and shorter shafts and semi-rounded, scooped lobes, exhibiting a moderate variety of morphology in the leaves of rice species [27]. Saddle phytoliths in bamboo leaf exhibited taxonomical value both at the subfamily and genus levels [11]. Saddle was also found in *Chusquea simpliciflora* [28] and rice species [26]. In this study, saddle phytoliths in the culm sheath bodies showed insignificant variation, implying that they were stable and minimally affected by different growth stages, and their parameters might have taxonomical value. *D. brandisii* culm sheaths were characterized by a high proportion of rondel phytoliths, which was similar to that in crops and other grasses [24,25,26]. Rondels were first described in *Zea mays* [25] and were common to moderate in the inflorescences and leaves of domesticated and wild rice species [24,26]. Most rondels in bamboo species exhibited distinctive three-dimensional structures but not genus-specific forms, since they overlapped with the Oryzoideae and Panicoideae [26,29]. In the present study, it was noted that the circular phytoliths occurred at relatively low frequency in the *D. brandisii* culm sheath blades. Similarly, circular phytoliths rarely exceeded 1% of the total phytoliths in the foliage leaf blades [12]. Parallelepipedal bulliform cells contain square and rectangular (blocky) phytoliths, while cuneiform bulliform cells form fan (bulliform flabellate) phytoliths [26]. Cuneiform bulliform cells are abundant in woody bamboo species. The shape of cuneiform bulliform cells is considered as an effective criterion for determining sub-species of ancient rice [24,26]. However, the bulliform flabellate (fan) phytoliths observed in *D. brandisii* foliage leaf blades or other bamboo species [11,12] were not found as observed in the culm sheath blades. Since few bulliform cells were observed in the culm sheaths, the number of bulliform cells represented the significant difference between culm sheath and foliage leaf. In this study, the bulliform flabellate phytoliths were not observed in the *D. brandisii* culm sheaths, but blocky phytoliths were observed with high occurrence. Most bamboos have their stomata on the abaxial surface of their foliage leaf blades. In contrast, more stomata were found on the adaxial than the abaxial epidermis in the culm sheaths of *F. yunnanensis* [1]. The stomatal density of culm sheaths was far less than that of the foliage leaf blades [1]. However, the stomata phytoliths were observed in the culm sheaths (>10%) in our research but were not being observed in the *D. brandisii* foliage leaf blades [12]. The culm sheath was the main transpiration and respiration organ of bamboo shoot through a large number of stomata and high activities of transpiration and respiration [4]. The stomata of the foliage leaf blades were usually closed and covered by four papillae in the abaxial epidermis, whereas, in the culm sheaths, they were open and had no papillae. Therefore, the stomata were not as easily observed because the papillae overlay the stomatal cells in foliage leaf blades [1]. That is why a certain proportion of stomata phytoliths were observed in the *D. brandisii* culm sheaths, but were not observed in the foliage leaf blades. The phytolith morphotypes of elongate and acute were more frequently reported (>10%) in the *D. brandisii* culm sheath blades. Elongate phytoliths, originating from the long cells of the epidermis, were the dominant phytolith morphotype in bamboo branches [16] and were abundant to common in the leaves and inflorescences of rice species [26], whereas in the foliage leaf blades, they occurred only at relatively low frequency (less than 4%) [16].

### 3.3. Evolution of Phytolith Morphotypes with Sheath Growth and Function

Previous studies showed that phytolith morphology is associated with the cell structure and function of different organs [11,12,16]. The bamboo sheath completely surrounds and protects new shoots, playing a crucial role in maintaining culm stability during development and growth [6,20]. Typically, elongate phytoliths, which form in the long cells of vascular tissue, contribute to the water conduction, nutrient storage, and mechanical support in plants [30]. Elongated phytoliths enhance plant stiffness, thereby improving support for the plant [9]. These elongate phytoliths were particularly abundant in *D. brandisii* culm sheaths, and their abundance increased as the sheath matures. Additionally, their size also increased with the culm sheath maturity. The higher ratio and greater size of elongate phytoliths in the culm sheaths enable phytoliths to hold together and play a skeletal role in bamboo plants [9], so as to better support and protect the bamboo shoots. Acute phytoliths, which mainly form in trichome cells and papillae [11], are instrumental in defending against vertebrate and invertebrate herbivores [31]. Silicon deposition, as a physical barrier, enhance the overall mechanical strength and external protective layer, thereby hindering fungal invasion and insect chewing, and protecting shoot growth. The high proportion of acute phytoliths in *D. brandisii* culm sheaths might be contribute to its enhanced ability to protect shoots from being damaged by fungi and herbivores. Specifically, the size of acute phytoliths in culm sheath blades at the shooting stage was the greatest, further enhancing its protective role on bamboo shoots.

Bamboo culm sheaths have physiological functions [4,5]. Since bamboo shoots and young culms have no branches, culm sheaths are the main transpiration organs. In *F. yunnanensis*, the transpiration rate and the stomatal conductance of culm sheaths were far higher than that of foliage leaf sheaths [4]. Originating from the motor cells of leaves, parallelepipedal and cuneiform bulliform cells are common in the grass family [12]. Parallelepipedal bulliform cells contain square and rectangular (blocky) phytoliths [12]. Generally, reducing the swelling of the bulliform cells that resulted from water loss may lead to the deposition of silica in these cells. It is believed that the more plants transpire and/or suffer water stress, the more silicified parallelepipedal bulliform cells they will produce [32]. Hence, blocky phytoliths were found in the *D. brandisii* culm sheaths with high occurrence. Moreover, the size of blocky phytoliths increased with culm sheath maturity. The sheaths at culm and shedding stages had blocky phytoliths with the greatest length and width values, implying that the transpiration of culm sheaths continued to strengthen with maturity. Moreover, the result also showed that the base part of the culm sheath bodies had higher blocky phytolith occurrence with bigger sizes than the middle and upper parts, indicating that the base part of the sheaths might have stronger transpiration ability.

Silica precipitates in tissues with photosynthesis activity [22] as a possible result of Si supersaturation due to boundary layer water loss via stomata [33]. It was reported that the culm sheath was the main transpiration and respiration organ for bamboo shoots and young elongating culms; the transpiration rate and stomatal conductance of culm sheaths were significantly higher than those of foliage leaf sheaths [4]. The silicon gradually accumulated in stomata, which led to the formation of stomata phytoliths. Therefore, the culm sheaths, undertaking respiration function, had a higher proportion of stomata phytoliths. This also confirmed the fact that the culm sheaths had physiological functions in addition to the function of mechanical support. Furthermore, the size of the stomata phytoliths decreased with the culm sheath maturity. The stomata phytoliths in the culm sheath blades at the shooting stage were bigger than those at the culm and shedding stages, implying that the culm sheath blades at the shooting stage might have a much higher respiration rate.

## 4. Materials and Methods

### 4.1. Materials

**Figure 6:**
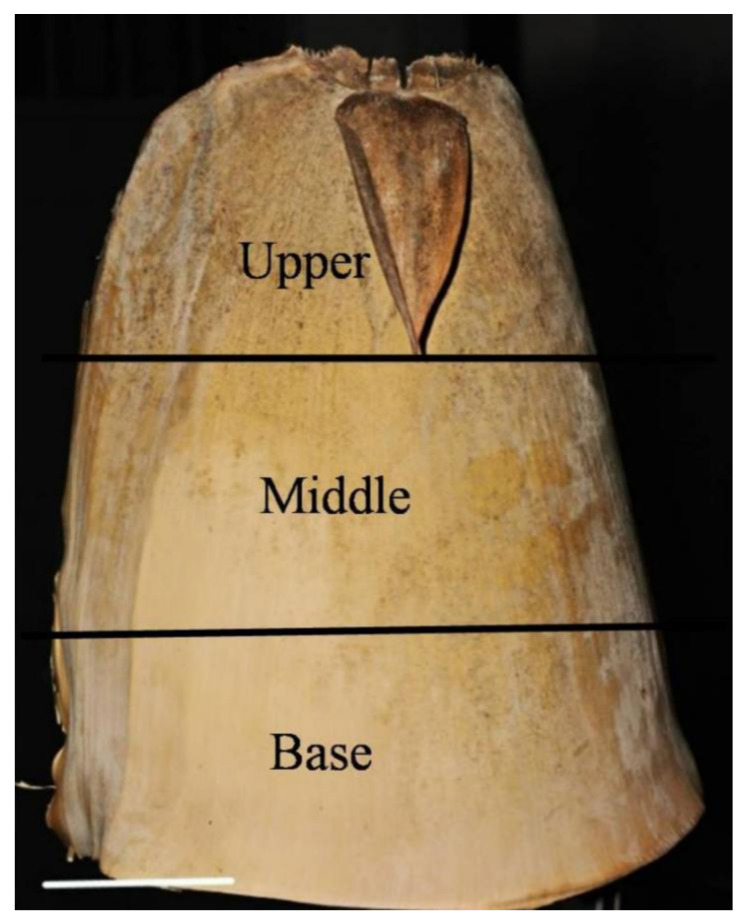
Culm sheath of *Dendrocalamus brandisii* (bar = 6 cm).

*D. brandisii* culm sheaths were collected in August 2023 from the bamboo garden at Southwest Forestry University in Yunnan Province of China (25°03′42″ N,102°45′40″ E; altitude:1850 m; annual average temperature: 16.5 °C and average rainfall: 1450 mm; soil: brown-red soil with pH 5~5.6). Nine sheaths, including three sheaths at three different growth stages from three bamboo groves, were collected, viz. culm sheath at shooting stage, tender, light brown, and tightly enclosing 2-week-old shoots (approximately 30 cm high); culm sheath at culm stage, hard, fragile, brown, and embracing the internodes of 1 to 3-month-old culm; culm sheath at shedding stage, hard and dark brown, fallen on the ground from 12 month culms. The culm sheaths were evenly divided into three portions from the bottom to the top, the base, middle and upper portions with the blades removed (Figure 6).

### 4.2. Determination of Silicon and Phytolith Content

Sheath samples were oven-dried at 60 °C for more than 24 h until completely dry and then ground in a Wiley mill. Ground material passing a no. 40 mesh sieve shaker but retained on no. 60 mesh was used to determine silicon content using the molybdenum blue spectrophotometric method described by Zou [34]. Phytoliths were extracted by chemical oxidation, dried in the centrifuge tube and weighted.

### 4.3. Phytolith Extraction and Observation

We extracted the phytoliths in the samples using chemical oxidation and slide-mounted the samples in Canada balsam as described in Pearsall [35]. A minimum of 3 slides were produced for each sample. At least 400 phytolith grains per slide were identified and counted, and the occurrence (%) of each morphotype was calculated following the morphological classification established in the International Code for Nomenclature of Phytoliths (ICPN) 1.0 [18] and ICPN 2.0 [19]. Each phytolith morphotype was photographed, and the morphological parameters were measured under a microscope (Leica DM 1000, Tokyo, Japan) to obtain data for horizontal width (W, width) and vertical length (L, length) with objective lenses at 40×, following the measurements by Wang and Lu [11] for phytolith size.

We analyzed the data obtained and compared by one-way ANOVA using the least significant difference method (LSD) to assess the significance at *p* < 0.05.

## 5. Conclusions

The silicon accumulation in the *D. brandisii* culm sheaths at different growth stages was comparable to leaves but significantly greater than branches in previous research. The phytolith concentration in the *D. brandisii* culm sheath blades was higher, which was associated with their greater silicon content than the culm sheath bodies. Silicon precipitated and phytoliths were produced as the sheath matured. The upper part of the culm sheaths at the shedding stage had significantly greater silicon and phytolith content. *D. brandisii* culm sheaths were characterized by a high proportion of rondel phytoliths, whereas bilobate phytoliths were not detected, and the saddle phytoliths were present with a very low proportion, which was different from that of foliage leaves. Bulliform flabellate phytoliths observed in the *D. brandisii* foliage leaves were not found in the culm sheaths. Stomata phytoliths were abundant in the culm sheaths and increased with sheath maturation, but were not observed in the foliage leaves. The elongate and acute phytoliths were extremely abundant in the culm sheaths and increased with sheath maturation, whereas in the foliage leaves, they occurred only at relatively low frequency. Circular phytoliths occurred at relatively low frequency in the culm sheaths, which was similar to foliage leaves. The higher ratio and greater size of elongate phytoliths in the sheaths at the culm stage indicated their mechanical support, while the higher proportion and greater size of acute phytoliths in the sheaths at the shooting stage might imply their protective role to resist fungal and herbivores. The increase in the ratio and size of blocky phytoliths with maturity implied the transpiration of culm sheaths continued to strengthen. A higher proportion of stomata phytoliths in the culm sheaths at the shooting stage supported the transpiration, respiration and photosynthesis in culm sheaths of the shoots. The phytolith morphotypes in the *D. brandisii* culm sheaths did not demonstrate a specific trend with position. Saddle phytoliths in the culm sheaths showed insignificant variation. The phytolith morphotypes and presence frequency in the *D. brandisii* culm sheaths varied in close association with its mechanical and physiological function.

## Figures and Tables

**Table 1:** Silicon and phytolith content in the *D. brandisii* culm sheath blades at different growth stages (g·kg^−1^).

Stages	Silicon	Phytolith
Shooting	32.92 ± 8.05 b	25.53 ± 1.38 c
Culm	127.18 ± 6.12 b	40.38 ± 23.33 b
Shedding	150.55 ± 2.72 a	103.44 ± 3.03 a
Means	103.55	56.45

Note: Lowercase letters in the same column denote the statistical difference among different sheath stages at *p* < 0.05 according to LSD.

**Table 2:** Silicon and phytolith content in the *D. brandisii* culm sheath bodies at different growth stages (g·kg^−1^).

Stages	Position	Silicon	Phytolith
Shooting	Upper	21.97 ± 1.97 a	16.21 ± 5.33 b
	Middle	25.38 ± 2.73 a	23.84 ± 2.75 a
	Base	23.92 ± 0.61 a	23.48 ± 1.76 a
	Mean	23.75 ± 1.71 B	21.18 ± 4.31 B
Culm	Upper	32.44 ± 2.27 a	27.45 ± 1.05 a
	Middle	31.37 ± 6.33 a	26.39 ± 3.38 a
	Base	39.41 ± 7.46 a	26.60 ± 7.93 a
	Mean	34.41 ± 4.37 A	26.81 ± 0.56 A
Shedding	Upper	38.05 ± 5.21 a	30.92 ± 2.58 a
	Middle	25.44 ± 4.64 b	22.90 ± 2.30 b
	Base	33.27 ± 1.50 ab	23.10 ± 2.69 b
	Mean	32.25 ± 6.37 A	25.96 ± 4.57 A
Means		30.14	24.54

Note: Capitalized letters in the same column denote the statistical difference among different sheath stages at *p* < 0.05 according to LSD. Lowercase letters in the same column denote the statistical difference at different positions of culm sheath bodies in each stage at *p* < 0.05 according to LSD.

## Data Availability

The datasets generated during and/or analyzed during the study are available from the corresponding author upon reasonable request.

## Supplementary Materials

The following supporting information can be downloaded at https://www.mdpi.com/article/10.3390/plants14060841/s1: Table S1. Length and width of different phytolith morphotypes in the *D. brandisii* culm sheath blades at different growth stages. Table S2. Length and width of different phytolith morphotypes in the *D. brandisii* culm sheath bodies at different growth stages.
